# Xenotransplantation of Human Neural Progenitor Cells to the Subretinal Space of Nonimmunosuppressed Pigs

**DOI:** 10.1155/2011/948740

**Published:** 2011-06-01

**Authors:** Karin Warfvinge, Philip H. Schwartz, Jens Folke Kiilgaard, Morten la Cour, Michael J. Young, Erik Scherfig, Henry Klassen

**Affiliations:** ^1^Department of Ophthalmology, University Hospital of Lund, 22185 Lund, Sweden; ^2^Children's Hospital of Orange County, Orange, CA 92868-3874, USA; ^3^Eye Deptartment and Eye Pathology Institution, Rigshospitalet, 2100 Copenhagen, Denmark; ^4^Copenhagen Ophthalmology Center, Glostrup Hospital, 2600 Copenhagen, Denmark; ^5^Harvard Medical School, Schepens Eye Research Institute, Boston, MA 02114, USA; ^6^Stem Cell Research Center, Sue and Bill Gross Hall, University of California, Room 2035, 845 Health Sciences Road, Irvine, CA 92697-1705, USA

## Abstract

To investigate the feasibility of transplanting human neural progenitor cells (hNPCs) to the retina of nonimmunosuppressed pigs, cultured hNPCs were injected into the subretinal space of 5 adult pigs after laser burns were applied to promote donor cell integration. Postoperatively, the retinal vessels appeared normal without signs of exudation, bleeding, or subretinal elevation. Eyes were harvested at 10–28 days. H&E consistently showed mild retinal vasculitis, depigmentation of the RPE, and marked mononuclear cell infiltrate in the choroid adjacent to the site of transplantation. Human-specific antibodies revealed donor cells in the subretinal space at 10–13 days and smaller numbers within the retina on days 12 and 13, with evidence suggesting a limited degree of morphological integration; however, no cells remained at 4 weeks. The strong mononuclear cell reaction and loss of donor cells indicate that modulation of host immunity is likely necessary for prolonged xenograft survival in this model.

## 1. Introduction

Neurological disorders comprise a wide spectrum of conditions affecting all parts of the central nervous system (CNS), including the brain, spinal cord, and retina. These diseases are common, often debilitating, and generally recalcitrant to treatment. In an effort to generate novel approaches to CNS repair, particular attention has been given to diseases of the retina where the biological challenges present are arguably more circumscribed, the existing surgical techniques notably precise, and the medical imaging and functional monitoring capabilities relatively advanced. Although mammals do not share the innate capacity for retinal regeneration displayed by many teleost, urodele, and anuran species, there is now a sizeable literature documenting the restorative potential of transplanted stem and progenitor cells in animal models retinal disease (as reviewed in [1]). 

The types of stem and stem-like cells that have been used as donor cells for retinal transplantation range from embryonic stem cell [2] and induced pluripotent stem (iPS) cells [3] to brain- and retina-derived CNS progenitor cells [4, 5], primary rod photoreceptor precursor cells [6], and bone marrow-derived populations such as vascular progenitors [7]. Gratifying results have been frequently reported, regardless of cell type, although here it should be noted that a number of caveats apply. Pluripotent cells typically require partial predifferentiation into lineage-committed progenitor cells prior to transplantation to improve the yield of desired mature cell type and to avoid teratoma formation. Photoreceptor precursors can be enriched from immature transgenic murine tissue, but the isolation of clinically significant yields of human precursors has not yet been possible such that the translation of this approach will likely require additional scientific advances. Currently, bone marrow and CNS progenitors are particularly attractive from the standpoint of preclinical development, and of these, the latter has the added advantage of exhibiting the capacity for neuronal cell replacement in the diseased retina. 

CNS progenitor cells have now been derived from the brain or the retina of multiple different mammalian species, including humans [8], and transplanted to the retina of the mouse [9], rat [4, 9, 10], Brazilian opossum [11], pig [12–14], cat [15], and monkey [16]. Donor cell survival has been consistently reported over a varying range of survival times. In none of these instances were the cells autologous, and in the majority of cases, the recipient animals did not receive immune suppression. The ability of allogeneic CNS progenitor cells to survive transplantation to immune competent hosts is robust and reproducible, but not invariant, as has been particularly well characterized in the mouse [17]. The apparent immune privilege status of CNS progenitors as donor cells is a factor that might enhance the clinical utility of these cells although an important caveat here is attention to treatment conditions that might influence expression of the major histocompatibility complex (MHC), particularly class II antigens [18].

In addition to allografting experiments, CNS progenitors have been transplanted to the vitreous and retina as xenografts. For instance, grafts of brain-derived GFP+ murine NPCs have been performed in the rat [10] and the Brazilian opossum [11], in both cases without immune suppression. In addition, GFP+ murine retinal progenitor cells (RPCs) have been transplanted to the subretinal space of the pig [12, 13]. Overall, integration and survival varied between models and appeared to depend largely on the degree of host immune reactivity to the grafts; however, it is important to note that the rejection of CNS progenitor xenografts was not invariable, and survival out to 4 weeks was possible in some instances. 

The availability of human NPCs [19, 20] and RPCs [8, 21] has increased the need for xenogeneic animal models for safety and efficacy testing of these cell types. Previous reports include studies in rat [9], monkey [16], and mouse [22]. Reported results typically included animals that were exogenously immunosuppressed or exhibited endogenous immune insufficiency, making the interpretation of immune tolerance difficult. Here, we investigated the xenotransplantation of brain-derived human NPCs to the subretinal space of nonimmunosuppressed pigs.

## 2. Materials and Methods

### 2.1. Donor Cells

Donated tissue was obtained under informed consent, and all work was performed with IRB approval (Children's Hospital of Orange County). The donor cells used in this study were derived from postmortem forebrain tissue obtained from an infant that was delivered prematurely at 25 weeks gestational age as has been described previously [20]. Briefly, tissue was harvested by dissection under semisterile conditions, minced in a tissue culture hood, and cultured in DMEM/F12-based growth medium supplemented with BIT 9500 (Stem Cell technologies, Vancouver) containing EGF (20 ng/mL) and bFGF (40 ng/mL) as mitogens, as well as PDGF-AB (20 ng/mL) to retain the potential for oligodendrocyte differentiation. Penicillin, gentamicin, ciprofloxacin, and amphotericin were also included to avoid the contamination of primary cultures. After expansion in fibronectin-coated tissue culture flasks for approximately 6 weeks, the resulting cell culture used in the present study was designated SC27 [20]. 

### 2.2. Animal Recipients

Host animals were 5 juvenile female pigs of the Danish landrace, 4 months of age, and approximately 30 kg in weight. Surgery was performed in one eye only (left) under general anesthesia. No immunosuppressive drugs were administered. All animal work was performed under the approval of the supervisory authorities of the Panum Institute, University of Copenhagen and in accord with the ARVO policy for the treatment of animals.

### 2.3. Transplantation

Animals were placed under general anesthesia, and vitreoretinal surgery was performed as previously described [12]. Briefly, the pigs were pre-anesthetized with intramuscular injections consisting of midazolam, zolazepam, tiletamine, xylazine, ketamine, and methadone. They were then intubated, artificially ventilated, and anesthetized with isoflurane/oxygen. The operative pupil was dilated with topical phenylephrine, tropicamide, and atropine. The surgical field was prepared and draped in the usual sterile fashion before commencement of surgery. A localized 3-port pars plana vitrectomy was performed and green argon laser burns applied in a grid pattern to the area centralis of the retina. A retinotomy was prepared and the human cells (approximately 1 × 10^6^) delivered via a 41-gauge needle to the subretinal space in the region corresponding to the laser burns. Correct graft placement was ascertained by direct visualization at the time of injection. Chloramphenicol was given prophylactically at the end of surgery to avoid infection.

### 2.4. Postsurgical Followup and Survival Times

Animals were followed clinically, including funduscopic examination on a weekly basis. Survival times were 10 days, 11 days, 12 days, 13 days, and 4 weeks, at which time the animals were placed under general anesthesia, and the previously treated eyes were enucleated and processed for histological analysis. Following enucleation, animals were terminated using intravenous sodium pentobarbital, as previously described [12].

### 2.5. Histology

Enucleated globes were fixed by immersion in 4% paraformaldehyde and processed for histology as previously described in detail [12]. Briefly, globes were immersion fixed in 4% paraformaldehyde for 10–20 minutes. The anterior segment, including lens, was then removed, and the posterior segment was postfixed for an additional 2 hours, again in 4% paraformaldehyde. A tissue block including the area of interest, optic nerve head, and temporal periphery was prepared and embedded in gelatin, and serial 12 *μ*m sections were cut by cryostat. A subset of sections (1 : 10) was stained with hematoxylin-eosin (H&E), and the remainder was reserved for immunohistochemical labeling. 

### 2.6. Immunohistochemistry

Sections were processed with primary antibodies (Table 1) in a moist chamber at 4°C for 16–18 hours, followed by rinsing in PBS/Triton X-100, prior to labeling with secondary antibodies (Table 1) for 1-2 hours in the dark at room temperature, as previously described [12, 14]. Negative controls with primary antibodies omitted were also performed. Sections were imaged through an epifluorescence microscope.

## 3. Results and Discussion

A total of 5 pigs received subretinal xenografts of human neural progenitor cells. Of these, 4 pigs were sacrificed after 10–13 days and 1 pig after 28 days. Results of the antihuman immunolabeling were most consistent with the antinuclei antibody (Table 1). Using this reagent, surviving human donor cells were identified in the initial 4 animals, but not in the last (Table 2). The amount of surviving cells varied between recipients, as did the distribution of cells in host tissues; however, a number of points can be derived from these data. 

**Figure 1:**
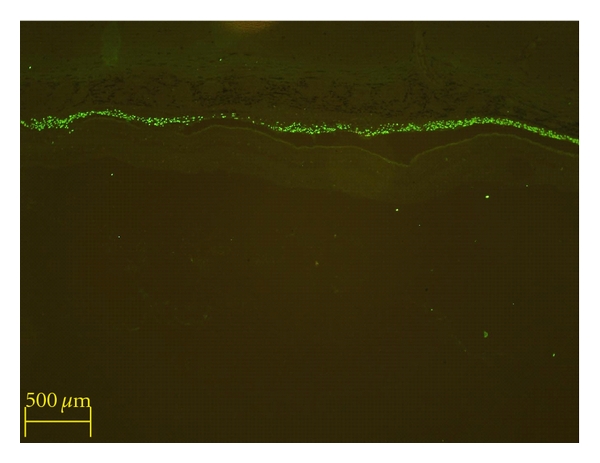
Identification of donor cells, graft placement, and survival at 10 days. Donor cells were identified via anti-human nuclear immunolabeling and an FITC-conjugated secondary antibody. Abundant human nuclear profiles (green) extend in a line horizontally across the image, the location of which is the subretinal space, corresponding precisely to the initial site of deposition. In this particular view, there is no evidence of migration or integration of donor cells into host tissues. The retina, seen here as the faintly striated, unlabelled tissue directly beneath the grafted cells, appears detached from the overlying structures; however, this was not seen in vivo and is an artifact of the method of histological processing employed.

It is clear that hNPCs can survive in the subretinal space (SRS) of nonimmunosuppressed pigs for a minimum of 13 days. The immunohistochemical results also back up the observations made at the time of surgery regarding effective placement of the grafts in the subretinal space (Figure 1). In all cases in which surviving human NPCs were present, a portion were located in the subretinal space, and these represented the majority of the cells. In addition, there was a widespread lateral distribution of the hNPCs within the host SRS, tending to confirm the clinical observation that the retinal bleb formed at the time of injection had resolved with even distribution of the grafted cells in the SRS, despite later artifactual detachment of the neural retina during tissue processing (Figure 1).

**Figure 2:**
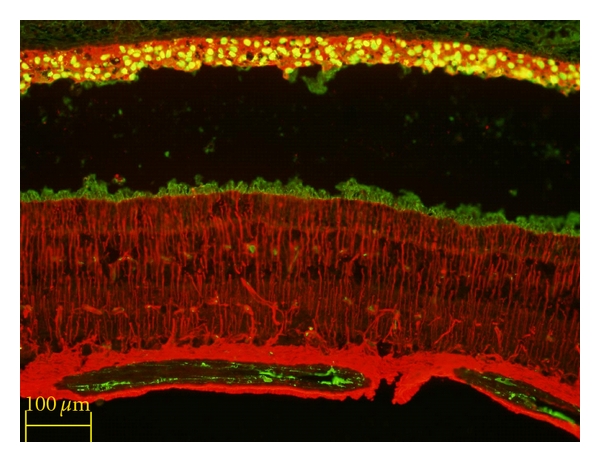
Xenografted human NPCs express GFAP in the porcine subretinal space. Donor cell identified with anti-human nuclear antibody exhibits cytoplasmic labeling for the astroglial marker glial fibrillary acidic protein (GFAP, red), which is also expressed by activated glial cells in the underlying host neural retina. Survival = 10 days.

**Figure 3:**
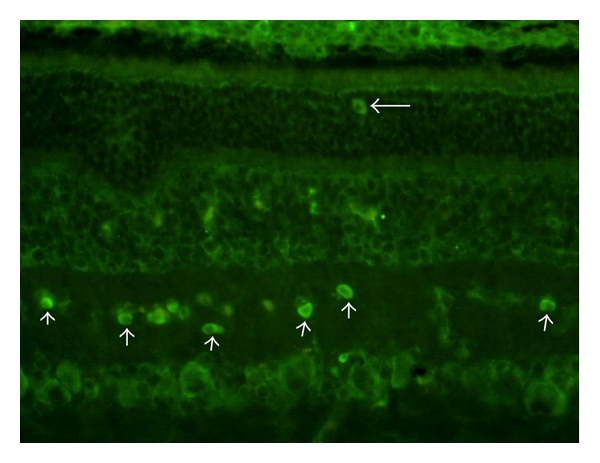
Xenografted human NPCs within the porcine retina. Donor cells were identified via anti-human nuclear immunolabeling and FITC-conjugated secondary (green). In addition to abundant cells within the subretinal space (top of image), labeled profiles were also evident within specific layers of the host retina, including the outer nuclear layer (horizontal arrow) and the inner plexiform layer (upward pointing arrows). In the latter case, the donor cells appear to be specifically positioned within a relatively restricted sublaminar zone. Survival = 12 days.

The donor cells remaining in the SRS strongly expressed the glial marker GFAP, suggesting the possibility of a predominant differentiation along astroglial lines by these cells (Figure 2). GFAP was also expressed in the host retina, likely as a reaction to prior surgical intervention, including the application of laser burns. It is also clear that donor hNPCs were capable of migration into adjacent host tissues over the restricted time course of this study. There was evidence of donor profiles within the neural retina, and these profiles tended to exhibit nonrandom localization, favoring (but not limited to) sites of prior laser application and showing preference for certain host cytoarchitectural landmarks, such as the inner plexiform layer (IPL; Figure 3). Although nuclear labeling does not allow the examination of donor cell morphology, in the example just cited the relative positional organization of the donor nuclei appears to reflect cytoarchitectural cues to the extent that there is sublaminar positioning within the central zone of the IPL, as opposed to the boundary regions of that layer. This is suggestive of at least some degree of morphological integration; however, no further conclusions regarding cell fate can be drawn. 

**Figure 4:**
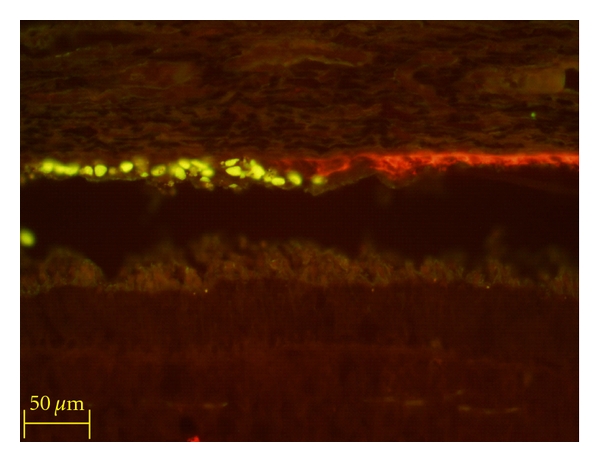
Xenografted hNPCs incorporated into the porcine RPE monolayer. Donor cells are labeled with anti-human nuclear label (upper left, yellow-green). In this case, a single donor profile is seen within the subretinal space, and the remainder are located at the level of the retinal pigment epithelium (RPE), labeled here with anticytokeratin (red). The human donor cells occupy a region corresponding to a focal discontinuity in the cytokeratin labeling. Survival = 10 days.

Additional evidence of donor cell integration is provided by the examination of the retinal pigment epithelium (RPE). Immunolabeling indicates the migration of hRPCs from the SRS into circumscribed regions of the RPE monolayer, with some degree of morphological integration (Figure 4). The donor cell nuclei localize precisely to regions where a gap is present in the host RPE monolayer, as evidenced by discontinuity of cytokeratin labeling. It would appear that the donor cells have been attracted to an area of laser injury and have reformed a donor-derived monolayer in response. Note that this “repair” is not perfect: there is a degree of disorganization at the junction between graft and host monolayers, as well as a lack of cytokeratin expression by the host cells. The hRPCs have therefore been not differentiated into mature RPE cells, and therefore, this situation does not comprise functional integration, neither does it rule out the possibility should longer survival times be obtainable. Interestingly, very similar results were seen following xenografts of murine retinal progenitor cells (RPCs) to the porcine SRS [12]. 

**Figure 5:**
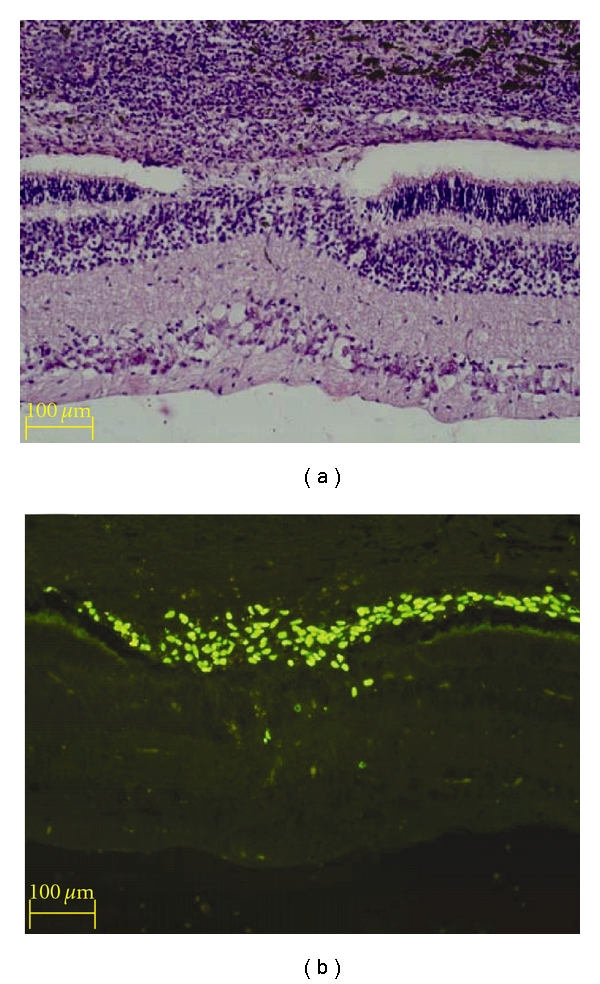
Evaluation of host tissue and cellular response in the area of laser burn. A subset of sections from eyes with xenografts were stained with H&E (a) to evaluate the host tissue and cellular responses, while adjacent sections were immunolabeled to identify human donor cells (b). (a) The area of laser injury can be identified by the focal disturbance of the overlying pigmented layers, as well as localized disruption of the underlying retina. An abnormal adhesion between the retina and overlying structures is present in this location that bridges the subretinal space. A dense mononuclear infiltrate is present in the choroid (top), consistent with the development of a strong immune response. (b) Immunoimaging reveals the presence of many donor cells, which have preferentially congregated in the area of laser injury and appear to contribute to the subretinal adhesion. A few donor profiles are present in the neural retina in the immediate vicinity of the laser injury. Survival = 10 days.

Finally, it is clear that human-to-pig xenografts evoke a powerful immune response, even when placed within the immunologically privileged confines of the SRS (Figure 5). The rejection response seen is disproportionally choroidal in origin, with little evidence of retinal involvement until late in the process, likely explaining the preservation of donor cells at the early time points, when the choroid already contained a cellular infiltrate. Both the timing and pathological features of this response are quite similar to those already documented for mouse-to-pig xenografts [12, 13]. In either case, survival out to 2 weeks is obtainable without host immune suppression, whereas loss of the graft was invariable at 4 weeks. An important but unresolved issue concerns the potential role of laser treatment in the inflammatory response seen here and in the prior studies. Laser application serves to increase donor cell integration yet at the same time leads to focal injury of the RPE and likely alters the blood-retinal barrier. Thus, laser burns could exacerbate an underlying lack of tolerance for xenogeneic cells. In contrast, 4-week donor cell survival without immunosuppression was seen following xenotransplantation of mouse NPCs to the eye of the early postnatal Brazilian opossum [11]. Choroidal infiltrates were not seen in monkeys that received hNPC xenografts although varying degrees of immune suppression were used in that study [16]. Therefore, it appears that the porcine immune system represents a particularly formidable barrier to long-term xenograft survival.

## 4. Conclusions

The pig represents a large animal model of considerable recent interest for the development of novel surgical treatments, including stem cell transplantation. In previous work, we have shown that allogeneic grafts of CNS progenitor cells are well tolerated in the porcine retina, subretinal space, and vitreous cavity in the absence of immune suppression. However, we have also shown that xenografted murine CNS progenitors are rapidly rejected under similar conditions [12] and that the nature of the rejection response points to mouse and pig being immunologically discordant species [13]. Here, we show that human neural progenitor cells can integrate into the porcine retina; however, survival is limited to a brief window of approximately 2 weeks, beyond which an intense cellular response destroys the graft with considerable effacement of adjacent host tissues. These results are quite similar to our previous findings with mouse-to-pig xenografts. Even during the first 2 weeks, while human donor cells were surviving, intense hypercellularity was already evident in the adjacent choroid at the earliest time point examined (10 days), indicating a host cellular response to the graft. Of note, the reaction was elicited using a relatively well-tolerated cell type that was placed in a location known to exhibit aspects of immune privilege. Since both of these factors should tend to mitigate immunological responsivity, it can be anticipated that the outcome would not be better, and likely worse, following xenotransplantation of more immunogenic human cell types (e.g., those with prominent MHC class II expression) to conventional graft sites (i.e., lacking immune privilege). With these limitations noted, it may still be the case that substantially longer survival of human-to-pig xenografts could be obtained under conditions in which the host immune response is diminished, for instance, via exogenous suppression, innate insufficiency, or host humanization. Given the potential utility of the pig in translational development of regenerative therapies, it would seem worthwhile to further explore this possibility, despite the challenges faced.

## Figures and Tables

**Table:** (a) Antihuman primary antibodies

Antigen	Isotype	Company, product no.	Dilution
Human tau	Mouse monoclonal	Abcam, ab67360	1 : 100
Human nuclei	Mouse monoclonal	Chemicon, MAB1281	1 : 100
Human NCAM	Mouse monoclonal	Immunkemi, NCCD561B6	1 : 50

**Table:** (b) Primary antibodies against lineage markers

Antigen	Isotype	Company	Dilution
GFAP	Rabbit polyclonal	DAKO	1 : 100
Cytokeratin	Rabbit polyclonal	DAKO	1 : 100

**Table:** (c) Secondary antibodies

Fluorophore	Company	Dilution
FITC conjugated	Jackson Immunoresearch	1 : 200
Texas red conjugated	Jackson Immunoresearch	1 : 200

**Table 2:** Semiquantitative assessment of graft survival per pig and time point.

Pig ID no.	Survival	Donor cells	Distribution
211	10 days	+++	SRS, retina, RPE
208	11 days	++	SRS, retina
202	12 days	+++	SRS, retina
213	13 days	+	SRS
207	28 days	−	

Abbreviations: +++: many cells present, ++: moderate numbers of cells, +: a few cells, −: no cells, SRS: subretinal space, RPE: retinal pigment epithelium.
